# Anne O’Tate

**DOI:** 10.5195/jmla.2017.92

**Published:** 2017-04

**Authors:** Keith D. Engwall

## INTRODUCTION

Anne O’Tate is an alternative interface for searching PubMed developed by the University of Illinois at Chicago (UIC). It was developed as part of the Arrowsmith project, which has been developing informatics tools for advanced text mining of the biomedical literature. The tool is hosted on the Arrowsmith website on UIC servers and is freely available to the public [1]. The tool is designed to mine results data for relevant keywords, Medical Subject Headings (MeSH) terms, and bibliometric data to help users refine and develop their search strategies.

## DESCRIPTION

Anne O’Tate interfaces with PubMed through an application program interface (API) developed by the National Center for Biotechnology Information (NCBI). Users enter search terms in a text field, and those terms are passed to PubMed through the API, which then returns the results set. A “Limits” tab provides a subset of PubMed search filters, and a “Details” tab displays the detailed search string that is passed to PubMed. Anne O’Tate’s intended audience appears to be librarians and researchers conducting a general literature search, particularly those having a difficult time refining their search strategies.

## FEATURES

### Basic search

The front page provides the user with a search box with Limits and Details tabs, a bulleted list of instructions, help text with links to various PubMed help pages, and a description of the tool with a link to an article outlining the tool and the algorithms used for its functions. Unlike PubMed, the search box does not provide any predictive suggestions, but otherwise the search text is treated the same as if the user entered it in PubMed. Natural language is mapped, and field tags and Boolean operators are recognized.

The Limits tab includes drop-down menus for Field, Publication Type, Age, Language, Humans and Animals, Gender, Publication Date, and PubMed subsets. There is also a checkbox to limit results to those that have abstracts. The options available in the filters represent a subset of the filters available in PubMed and cannot be customized. The Details tab displays the full search string used by PubMed and is the equivalent of the Search Details box in the PubMed interface. Selected Limits are displayed in bold at the top of both the Limits and Details tabs, but they do not display on the search results page.

### Search results

Search results are presented in reverse chronological order, twenty items per page, displaying a citation for each record along with its PMID and a link to related articles. The title links to the PubMed record, and each author name links to an Arrowsmith index that displays the full names of all authors that match the name, their years of publication, their affiliation information, and topics that they frequently publish about, with links to that author’s publications in both PubMed and Anne O’Tate. The Related Titles link pulls up the full list of similar articles from PubMed.

### Search refinement

Anne O’Tate does not perform any data mining on the initial results set. Instead, it provides a list of data mining tools in the left sidebar, each of which will perform a specific function. The first tool in the list, Important Words, will analyze the text in the title and abstract of each result for words that “show high enrichment” and “should have high coverage” [1]. To the best of my understanding, these concepts relate to the uniqueness of the word within the results set as compared to all articles and the frequency of the word within the results set, respectively. This calculation, based on an index of all words in the titles and abstracts in MEDLINE, is updated annually.

The Important Phrases tool uses TopMine, which performs “phrase mining based on raw frequency as well as document context” [2], to display a relevance-ranked list of phrases from titles and abstracts. Important MeSH Pairs provides a list of MeSH term pairs, sorted by the odds ratio (probability) that two terms are assigned to the same article. The Mine the Gap tool performs a gap analysis, looking for expected MeSH pairs in the results set. The Topics, Authors, Affiliations, and Journals tools provide simple frequency-sorted lists from the results set. The Author Count and Year tools provide a text-based histogram of articles in the results set, categorized by the number of authors per article and publication year, respectively. Finally, the Cluster by Topic function provides a list of clusters for the top fifteen MeSH terms, along with a Most Recent Articles cluster for recent articles that are not yet indexed and a Not Indexed by Topic cluster for older unindexed articles. Some articles from the results set that are not included in any of the above clusters are placed in a Miscellaneous cluster, presumably those that have been indexed but whose MeSH terms were not ranked in the Topics tool.

Selecting a data mining tool will open a new browser tab containing the results of the corresponding function. The initial results set will remain in its own tab. The results from these data mining tools can be used to refine the results, and using the tools against the new results set will yield new results.

## EVALUATION

The Anne O’Tate interface is very spare and unadorned, clearly prioritizing function over form. Simple tables are used to present the output of most of the data mining tools, and all visualizations, such as histograms, are displayed using ASCII text. Although initial search results are returned reasonably quickly, the data mining functions are very slow and degrade in performance in proportion to the size of the results set. The phrase and gap analysis tools are considerably slower than other data mining tools. The interface and performance make Anne O’Tate clunky to use.

On the other hand, the quality of the information yielded by the tools in Anne O’Tate should not be understated. The Important Words and Phrases tools provide excellent suggestions for relevant keywords in a search set. The MeSH Pairs reveals tight relationships between MeSH terms in the results set. The Authors and Journals tools can be used to identify topic experts and potential avenues for publication. Also, the Year tool can be used to show publication trends. These tools can be used iteratively as the results set is refined, helping the user quickly home in on a small set of highly relevant articles. The Affiliation tool is not as useful as the rest, perhaps because affiliation information that publishers provide tends to vary widely. Affiliations may take the form of a country, city, or department name, making it extremely difficult to derive any meaningful information from the Affiliation tool results.

Anne O’Tate’s results are highly accurate and verifiable. The Details tab allows the user to see the exact search used in PubMed. The user can use the Boolean AND on this search string with a field-specific refinement term (such as an author or journal title) in PubMed to confirm the results. The one caveat to this is that the internal index of MEDLINE words that are used in the “enrichment” calculations for the importance ranking is only updated annually, which can impact the ranking for recent highly published topics. It is unclear, however, just how much inaccuracy this produces, given that the “coverage,” also used in the importance ranking algorithm, is based on frequency calculations from the live results set.

## SIMILAR TOOLS

Alternative interfaces to PubMed are becoming increasingly difficult to find. Many of the tools listed on the PubMed Alternative Interfaces page of the Health Librarians wiki, HLWIKI International—such as GoPubMed, eTBLAST, and PubFocus—either no longer exist or do not seem to be maintained. Quertle, now Quetzal-info, requires a paid subscription to access. The two remaining tools on HLWIKI’s “Best of Breed” list are BibliMed and PubGet [3]. PubGet, a free PubMed interface, provides only a very rudimentary search interface and provides no way to refine your search, and thus is too dissimilar to Anne O’Tate for an adequate comparison.

BibliMed is a free PubMed interface (registration required) that provides a more robust set of features than PubGet. The initial search field provides some predictive MeSH suggestions, although these are not required. The BibliMed results page provides a relevance ranked list of MeSH terms along the left sidebar and a list of relevant books (retrieved from Amazon) on the right sidebar. Above the search results are links to numerous related resources, such as PubMed Health, Trip EBM search, Google Scholar, and ClinicalTrials.gov, to name a few. Biblimed provides various filters, for example, Study Type, Clinical Filters (similar to PubMed Clinical Queries), and Focus (treatment, process, outcomes, and so on).

Searches of BibliMed and Anne O’Tate provide the same results, although BibliMed does not let users see exactly what search is being passed to PubMed. There is overlap between the most relevant BibliMed MeSH terms and the top Anne O’Tate topics, but they are clearly using different algorithms. BibliMed also lets users list MeSH terms alphabetically and uses word-cloud-style font enlargement to indicate relative relevance. The two tools provide very distinct filters, with BibliMed’s more focused on clinical filtering and Anne O’Tate’s more focused on general literature search. BibliMed offers no additional data mining or search refinement options.

Whereas Anne O’Tate’s interface is arguably too basic, BibliMed’s interface suffers from an excess of inconsistent formatting. The results page is a jumble of icons; fonts of various sizes, colors and weights; and screen elements. It is jarring and difficult to look at.

Although it is currently unable to connect to PubMed and does not seem to be actively maintained, gopubmed was the most similar tool to Anne O’Tate. Its ontology-based search methods provided semantic analysis of the title and abstract text in the results set [4]. Gopubmed provided relevance-ranked MeSH topics as well as in-text annotation of mapped MeSH terms. Gopubmed also provided very similar author, journal, and publication date rankings to Anne O’Tate and, through its Statistics page, provided gorgeous and useful visualizations including histograms, a world map, a publication timeline, and an author network map. Its speed, clean interface, and visual tools would have given it a huge advantage over Anne O’Tate. It is worth looking at if only to see what a great PubMed tool could be.

## CONCLUSION

In spite of its obsolete look and feel and its slow performance, Anne O’Tate provides an excellent tool set for searching PubMed. Its data mining tools provide a variety of dynamic content analysis that can be of great use in identifying relevant search terms and bibliometrics. Its performance may prevent it from being a go-to tool for day-to-day searching, but for difficult or complex searches, Anne O’Tate may be just the supplementary tool for your PubMed toolbox.

## References

[1] Smalheiser NR, Zhou W, Torvik VI (2008). Anne O’Tate: a tool to support user-driven summarization, drill-down and browsing of PubMed search results. J Biomed Discov Collab [Internet].

[2] Liu J, Shang J, Wang C, Ren X, Han J Mining quality phrases from massive text corpora [Internet].

[3] PubMed alternative interfaces [Internet].

[4] Doms A, Schroeder M, Bry F, Maluszynski J (2009). Semantic search with GoPubMed. Semantic techniques for the web: the REWERSE perspective.

